# Retrospective analysis of the efficacy of total glycosides of paeony capsules combined with hydroxychloroquine in patients with primary Sjogren’s syndrome

**DOI:** 10.12669/pjms.41.5.11102

**Published:** 2025-05

**Authors:** Tianxiao Fu, Wenwen Lu, Danbin Wu, Guolin Wu

**Affiliations:** 1 Tianxiao Fu Department of Traditional Chinese Medicine, the First Affiliated Hospital, Zhejiang University School of Medicine, Hangzhou, China; 2 Wenwen Lu Department of Traditional Chinese Medicine, the First Affiliated Hospital, Zhejiang University School of Medicine, Hangzhou, China; 3 Danbin Wu Department of Traditional Chinese Medicine, the First Affiliated Hospital, Zhejiang University School of Medicine, Hangzhou, China; 4 Guolin Wu Department of Traditional Chinese Medicine, the First Affiliated Hospital, Zhejiang University School of Medicine, Hangzhou, China

**Keywords:** HCQ, pSS, Retrospective study, TGP

## Abstract

**Objective:**

In this retrospective analysis, total glucosides of paeony (TGP) were compared with hydroxychloroquine (HCQ) in the treatment of patients with primary Sjogren’s syndrome (pSS).

**Methods:**

In this retrospectively analysis, 157 patients with SS treated at the First Affiliated Hospital of Zhejiang University School of Medicine hospital between January 2020 and December 2023 were included. The analysis included the flow rates of saliva and tears before and after treatment in the control group and observation group, as well as levels of white blood cells (WB), hemoglobin (Hb), platelets (PLT), and immunoinflammatory indicators including erythrocyte sedimentation rate (ESR), C-reactive protein (CRP), and serum immunoglobulin G (IgG) levels, disease evaluation scores and occurrence of adverse reactions.

**Results:**

Clinical baseline data were not statistically significant after six months of treatment between the two groups (*P*>0.05). In the observation group, the efficacy of treatment was 93.65%, whereas in the control group, it was 81.67% (*P<*0.05). Blood routine indicators (WB, Hb, PLT) significantly increased in the observation group (*P*<0.05). A significant decrease in immunoinflammatory markers (ESR, CRP, IgG) was observed in the observation group, compared with the control group (*P*<0.05). Disease evaluation scores scores in the observation groups significantly decreased (*P*<0.05). Adverse reactions showed no statistically significant (*P>*0.05).

**Conclusions:**

In the treatment of pSS, TGP capsules and HCQ are combined to improve salivary and lacrimal gland function, suppress the release of inflammatory factors, repair damage to the blood system, control disease activity, show significant clinical efficacy, and have few adverse reactions.

## INTRODUCTION

Sjogren’s syndrome (SS) is a persistent autoimmune disorder, with a global prevalence of 0.01% to 3%.^1^ Common clinical treatments include artificial tears, artificial saliva, corticosteroids, hydroxychloroquine (HCQ), and methotrexate as immunosuppressants, with an increased incidence of adverse reactions with long-term use. HCQ is commonly used as an immunomodulatory drug for treating SS, generally used to treat patients with symptoms like fatigue, joint pain without severe systemic manifestations.^2^

Total glucosides of paeony (TGP) is recommended in Chinese guidelines for treating systemic symptoms and organ involvement in SS and has been shown to significantly alleviate the symptoms of xerostomia and keratoconjunctivitis sicca in patients with SS. However, there is a lack of sufficient clinical data to summarize the effects of combining TGP with other Western medicines in treating SS. This study aimed to examine the therapeutic effects of combining TGP with HCQ in patients with pSS.

## METHODS

We retrospectively analyzed clinical data from 157 pSS patients treated at the First Affiliated Hospital of Zhejiang University School of Medicine between January 2020 and December 2023. During the study, there were 10 patients who were not followed up on, three patients discontinued treatment halfway, five patients experienced difficult to correct cardiopulmonary function damage during treatment, seven patients had liver and kidney function damage, a female patient became pregnant, two patients used immunosuppressants midway for other autoimmune diseases, and six patients developed severe infectious diseases.

### Inclusion criteria:

- Adheres to the 2016 classification diagnostic criteria established by the American College of Rheumatology / European League Against Rheumatism (ACR / EULAR).
- Age: From 18 to 75 years old.
- The patient is informed and consents to join the study.

### Exclusion criteria:

- Allergic to the drug under study.
- The patient did not receive treatment with glucocorticoids, immunosuppressants or other related therapies before the treatment.
- The presence of other rheumatic diseases.
- Patients presenting with severe pathologies affecting the cardiovascular, pulmonary, neurological, hepatic, renal, and hematopoietic systems.
- Infectious disease.
- Pregnant, lactating women, patients with mental illness, or poor compliance.

### Ethical approval:

This research has received approval from the Ethics Committee of the First Affiliated Hospital at Zhejiang University School of Medicine. (Approval No: IIT20220065C-X1, Dated: May 17, 2023) and has obtained informed consent from all subjects.

### Control group:

Given routine western medicine treatment. Hydroxychloroquine sulfate tablets (Plaquenil, SPH Zhongxi Pharmaceutical Co., Ltd. China Pharmaceutical Registration Number: H19990263) were used, with an initial dose of 0.2g per dose, twice a day, and reduced to 0.1g per dose when symptoms did not improve.

### Observation group:

TGP (Pavlin, Ningbo Lihua Pharmaceuticals Co., Ltd. China Pharmaceutical Registration Number: H20055058) was added to the standard hydroxychloroquine sulfate tablets treatment at a dose of 0.6g per dose, three times a day. Multiple indicators were evaluated prior to the initiation of medication and subsequent to the completion of treatment courses.

### Observation indicators:

### Clinical therapeutic effect:

Markedly effect: improvement of symptoms and signs, with improvement of two or more laboratory indicators by ≥70%. Effective: improvement of main symptoms and signs, with improvement of two or more laboratory indicators by <70%. Invalid: improvement rate of symptoms and signs <30%, with no improvement or worsening of laboratory indicators. Laboratory indicators include white blood cells (White blood cell), hemoglobin (Hemoglobin), platelets (Platelets), erythrocyte sedimentation rate (ESR), C-reactive protein (CRP), and serum immunoglobulin G (IgG) levels. Total effective rate = (markedly effect + effective) cases/total cases × 100%

### Salivary flow rate:

Patients refrained from brushing teeth, eating/drinking, smoking within three hours prior to the test. After clearing the mouth and collecting saliva at the bottom of the mouth, they were instructed to spit into a collection container every 60 seconds for 10 minutes to calculate salivary flow rate (ml/min).

### Tear secretion:

A 5mm x 30mm Schirmer test strip was folded at 5mm from one end to form a right angle, and this folded end was placed in the outer 1/3 of the conjunctival sac of both eyes. The participants closed their eyes, and the test strip was removed to measure the wet length from the fold after five minutes.

### Laboratory indicators:

WB, Hb, PLT, ESR, CRP, and IgG levels were tested before and after treatment. Patients were in a fasting state during the early morning hours, and 5mL of peripheral venous blood was collected. Department of Clinical Laboratory at the First Affiliated Hospital of Zhejiang University School of Medicine do the analysis.

### SS disease assessment criteria:

The European League Against Rheumatism Sjögren’s syndrome Disease Activity Index (ESSDAI) is used to assess disease activity. The European League Against Rheumatism Sjögren’s Syndrome Patient Reported Index (ESSPRI) is used to assess subjective symptoms. Disease damage is assessed using the SS Disease Damage Index (SSDDI) scoring table. Furthermore, we present the Fatigue Severity Scale (FSS) as the recommended instrument for assessing chronic disease-related fatigue in clinical settings.

### Adverse reactions:

Record the occurrences of rash, itching, abdominal pain, discomfort, nausea, vomiting, and blurred vision in patients during treatment, tally the corresponding number of cases, and calculate the overall incidence rate.

### Statistical Analysis:

SPSS 22.0 was used to analyze the data. Continuous data were shown as mean ± standard deviation (x¯±s). Independent sample t-tests compared groups, while paired sample t-tests compared within-group data before and after treatment. Categorical data were presented as percentages (%), and the χ^2^ test was used. A *p-value* of less than 0.05 was deemed to indicate statistical significance.

## RESULTS

The study’s final dataset comprised 123 patients, and 63 individuals assigned to the observation group, with an average age of (55.23±8.18) years, BMI index of (23.93±2.04) kg/m², disease course of (16.65±1.76) months, and a total of 26 complications. The control group consisted of 60 patients, with an average age of (53.87±7.71) years, BMI index of (23.32±1.79) kg/m², disease course of (17.02±1.49) months, and a total of 24 complications. The baseline data between the two groups did not exhibit a statistically significant difference (*P*>0.05) (Table-I).

**Table-I:** An analysis of general data.

Items	Observation group (n=63)	Control group (n=60)	χ2/t	P
Gender (male/female)	47/16	40/20	0.935	0.333
Age (years)	55.23±8.18	53.87±7.71	0.948	0.345
BMI (kg/m²)	23.93±2.04	23.32±1.79	1.759	0.081
Course of disease (months)	16.65±1.76	17.02±1.49	-1.255	0.211
Complications			0.021	0.886
Interstitial pneumonia	7	5		
lymphoma	5	6		
autoimmune liver disease	4	4		
other connective tissue diseases	10	9		

From a therapeutic standpoint, the observation group demonstrated significant improvement in 34 participants, moderate improvement in 25 participants, and no improvement in four participants, resulting in an overall efficacy rate of 93.65%. In the control group, 25 participants demonstrated significant improvement, 24 participants showed effective outcomes, and 11 participants were deemed ineffective, resulting in an overall effectiveness rate of 81.67%. A statistically significant difference was observed between the two groups (*P*<0.05) (Table-II).

**Table-II:** A comparison of two groups’ clinical efficacy.

Group	n	Markedly effective	Effective	Invalid	Total effective rate (%)
Observation group	63	34(53.97)	25(39.68)	4(6.35)	59(93.65)
Control group	60	25(41.67)	24(40.00)	11(18.33)	49(81.67)
*χ^2^*					4.122
*P*					0.042

Prior to treatment, no statistically significant differences were observed in hematological parameters (WB, Hb, PLT) and immunoinflammatory markers (ESR, CRP, IgG) between the two groups (*P*>0.05). After a six-month treatment period, both groups exhibited significant improvements in WB, Hb, PLT, with WB and Hb reaching the normal reference range. The observation group exhibited a more pronounced increase, which was statistically significant (*P*<0.05). Following treatment, there was a significant reduction in ESR, CRP and IgG levels, with ESR in the observation group returning to the normal reference range. CRP and IgG in both groups reached the normal reference range, with a more significant decrease in the observation group, demonstrating a significant difference (*P*<0.05) (Table-III).

**Table-III:** A comparison of blood routine and immune inflammatory marker levels.

Time	Group	n	WB(×10^9/L)	Hb(g/L)	PLT(×10^9/L)	ESR(mm/h)	CRP(mg/L)	IgG(mg/dl)
Before treatment	Observation group	63	3.16±0.28	112.34±5.13	165.75±8.69	59.34±3.67	8.82±1.17	2715.37±16.83
	Control group	60	3.23±0.33	110.85±5.24	163.21±7.37	60.28±4.19	9.02±0.98	2719.12±15.06
	*t*		-1.271	1.593	1.744	-1.325	-1.025	-1.335
	*p*		0.206	0.114	0.084	0.188	0.307	0.184
After treatment	Observation group	63	5.74±0.65*	136.22±7.27*	189.28±7.13*	21.08±4.91*	2.83±0.55*	935.87±12.53*
	Control group	60	4.66±0.50*	125.80±5.98*	185.56±8.06*	27.93±3.64*	3.30±1.01*	999.46±12.31*
	*t*		10.292	8.657	2.714	-8.755	-3.226	-28.376
	*p*		<0.001	<0.001	0.008	<0.001	0.002	<0.001

* , P<0.001 when compared with the same group before treatment.

Saliva and tear flow rates in the observation group and control group did not differ statistically significantly before treatment (*P*>0.05). After six months of treatment, both the saliva flow rate and tear flow rate in both groups significantly increased, with a more significant increase in the observation group, and there was a statistically significant difference (*P*<0.05) (Table-IV). Prior to treatment, no statistically significant differences were observed in the disease assessment criteria (ESSPRI, ESSDAI, SSDDI, FSS) between the observation group and the control group (P>0.05). After six months, both groups experienced significant reductions in ESSPRI, ESSDAI, SSDDI, and FSS scores, with the observation group showing a notably greater decrease (P<0.05) (Table-V).

**Table-IV:** Comparison of salivary and tear flow rate.

Time	Group	n	Salivary flow rate(ml/min)	Tear flow rate (ml/5min)
Before treatment	Observation group	63	0.15±0.04	1.95±0.72
	Control group	60	0.14±0.05	1.98±0.56
	*t*		1.228	-0.257
	*p*		0.222	0.798
After treatment	Observation group	63	0.23±0.03*	2.57±0.75*
	Control group	60	0.19±0.01*	2.22±0.47*
	*t*		9.82	3.084
	*p*		<0.001	0.003

* , P<0.001 when compared with the same group before treatment.

**Table-V:** Evaluation of disease outcomes between the two groups.

Time	Group	n	ESSDAI score	ESSPRI score	SSDDI score	FSS score
Before treatment	Observation group	63	6.61±0.34	4.85±0.42	5.15±0.44	53.53±4.18
	Control group	60	6.55±0.16	4.93±0.26	5.08±0.72	54.78±3.71
	*t*		1.242	-1.263	0.654	-1.751
	*p*		0.217	0.209	0.514	0.083
After treatment	Observation group	63	2.05±0.38*	2.33±0.45*	3.28±0.43*	34.12±2.87*
	Control group	60	2.89±0.51*	2.52±0.17*	3.67±0.26*	35.39±3.24*
	*t*		-10.391	-3.068	-0.605	-2.304
	*p*		<0.001	0.003	<0.001	0.023

* , P<0.001 when compared with the same group before treatment.

During the two treatment groups, the observation group had one case of rash, three cases of abdominal discomfort, one case of nausea and vomiting, and one case of blurred vision, with a total incidence of adverse reactions of 9.53%. The control group had three cases of abdominal discomfort, two cases of nausea and vomiting, with a total incidence of adverse reactions of 8.33%. The two groups did not differ statistically (P>0.05) (Table-VI).

**Table-VI:** A comparison of two groups’ adverse reactions.

Group	n	Rash	Abdominal pain and discomfort	Nausea and vomiting	Blurred vision	Total incidence rate (%)
Observation group	63	1(1.59)	3(4.76)	1(1.59)	1(1.59)	6(9.53)
Control group	60	0(0.00)	3(5.00)	2(3.33)	0(0.00)	5(8.33)
*χ^2^*						0.054
*P*						0.817

## DISCUSSION

HCQ is frequently utilized in the management of autoimmune diseases and is recommended as a first-line therapeutic agent for SS according to the Clinical Practice Guidelines.^3^ Several clinical studies^4^,^5^ have shown that HCQ does not effectively intervene in the progression of SS. Therefore, we believe that finding a more effective drug or combination therapy to alleviate dry symptoms and regulate systemic immune responses is of significant clinical importance.

Immune inflammatory response plays an important role in many rheumatic diseases, such as rheumatoid arthritis and SS. During the course of SS, inflammation persistently damages the glands and impairs their secretion function.^6^ Our team’s previous animal experiments have shown that TGP can improve the pathological damage and saliva secretion of the submandibular gland in NOD mice with SS by upregulating aquaporin-5 in the submandibular gland.^7^ TGP also improved SS by affecting the balance of Th1/Th2 cytokines and reducing the expression levels of interferon-γ(IFN-γ) and apoptosis related proteins.^8^ In addition, we found that TGP can improve the gut microbiota structure of SS mice by increasing the growth of key beneficial bacteria, inhibiting dominant pathogenic bacteria, and increasing the diversity and richness of gut microbiota, especially when combined with HCQ.^9^ Based on these previous studies, experimental evidence has been provided for us to choose the combination of HCQ and TGP. A multicentre, randomized, double-blind study in China showed that TGP can alleviate symptoms such as dry mouth and eyes in adult SS patients with a disease course of more than 24 weeks, and compared with the control group, ESSPRI, ESSDAI, and ESR showed a higher degree of improvement.^10^ Feng’s systematic review and meta-analysis indicated that TGP alone can improve tear secretion in patients, while in combination with immunosuppressants, it can enhance salivary flow rate, Schirmer test, and inflammatory markers (ESR, CRP, IgG, IgA, and IgM), which is similar to the immunoinflammatory markers observed in our study.^11^ The observation group in our research achieved a clinical efficacy rate of 93.65% after adding TGP, surpassing the control group, indicating that TGP as an adjuvant therapy for SS, when used in conjunction with other medications, controls the dysregulated immune-inflammatory response and enhances clinical efficacy.

This study found that the average values of blood cells in both groups were significantly decreased prior to treatment, below the normal range for white blood cells, at the lower limit of the normal range for hemoglobin, and within the normal range for platelets. After treatment, both groups reached the normal range, with the treatment group showing more significant improvement, which is related to the regulation of immune function by TGP, inhibition of B cell hyperactivity, and reduction in autoimmune complex formation. By comparing ESR, CRP, and IgG levels, we found a positive correlation between blood cell destruction and immune inflammatory factors, similar to Dai’s research.^12^ Overseas reports indicate that 30% of patients with SS have blood system damage, manifested as a reduction in one or more types of blood cells, with leukopenia being the most common.^13^ In a cross-sectional study of 132 SS patients, the prevalence of anemia was 34.1%, and was associated with patients’ ANA and anti-Ro/SSA.^14^ Anemia caused by pSS is often immune-mediated chronic disease anemia, while the decrease in platelets and white blood cells is related to immune dysfunction in the body, elevated levels of autoantibodies, inhibition of granulocytes, and destruction of peripheral blood cells by immune complexes. HCQ and TGP can inhibit such immune responses and exert the action of cortisol like hormones to restore blood cell damage.^15^

ESR, CRP, IgG are commonly used reference indicators for evaluating the body’s immune and inflammatory status, and are often used as markers for the development and activity of SS.^16^ This study found that before treatment, SS patients were in a state of high immune response, with ESR, CRP, and IgG levels all exceeding the normal range. After treatment, except for slightly high ESR in the control group, the other inflammatory indicators all reached the normal range. This indicates that HCQ and TGP can inhibit sustained inflammation by various mechanisms, such as suppressing excessive activation of T and B cells, restoring the damaged exocrine gland function, and the anti-inflammatory effect is better when TGP is used in combination. In a research report on saliva ultrasound elastography, it was found that serum ESR, IgG is a common reference indicator reflecting the severity of SS disease, which can quantitatively judge the progression of SS disease.^17^ ESSDAI is the gold standard for evaluating the activity of SS and is used as the primary outcome measure for the overall clinical activity of most SS patients. ESSPRI primarily evaluates dryness, pain, and fatigue symptoms in patients, complementing with ESSDAI. Considering the onset of SS from relatively stable or chronic progression to severe extraglandular system involvement, we lead into the SSDDI score.^18^ A retrospective study on SS in Japan demonstrated that the ESSDAI score is related to treatment intensity and is very helpful in evaluating drug efficacy and adjusting treatment strategies when using conventional drugs, immunosuppressants, and biologics.^19^ An early retrospective study in the Netherlands found that most patients showed low ESSDAI activity at the initial and final visits, with 1/4 of patients having ESSDAI ≥ 4, which is similar to our research results.^20^ We found that the vast majority of patients had an initial ESSDAI score of about 6 at their first visit, which could be reduced to two to three after treatment.

We found that there is a correlation between changes in blood systems, immune-inflammatory index, and ESSDAI, ESSPR, SSDDI, FSS in patients. Before treatment, the disease activity of SS was high, the subjective and objective rating indexes scores were high, the inflammatory markers increased significantly and exceeded the normal range, and there were also varying degrees of decreases in blood system. After treatment with HCQ alone and combined application with TGP, all indicators were significantly relieved. Clinically, improvements can be seen in saliva, tear time, and patient fatigue rating (FSS) after treatment, and there were significant differences in the improvements of various indicators after using TGP in combination. This is similar to the treatment outcome of a single center cross-sectional study conducted in India in 2022.^21^ Furthermore, the incidence of adverse reactions did not differ between two groups. This study adds relevant evidence to the medical literature on the treatment of SS with HCQ combined with TGP. Due to the difficult clinical treatment of SS and the significant side effects of hormones and immunosuppressants, the effect is not satisfactory, resulting in prolonged dry symptoms and lack of confidence in treatment. The appearance of TGP reduces the immune response of SS and has varying degrees of relief in disease activity, which is of great significance for clinical treatment. Although some studies have shown the potential therapeutic effects of TGP, there are still some aspects that need further investigation, including the pharmacological mechanisms of TGP in treating Sjogren’s syndrome, its biological effects on the body, and how it regulates the immune system and inflammatory response. As a component of traditional Chinese medicine, further evaluation of the safety of long-term use of TGP is needed, including potential adverse reactions and drug interactions. TGP, as an alternative therapy, requires comparative studies with traditional treatment methods or other drugs to evaluate its relative efficacy and advantages.

### Limitations & Strengths:

Firstly, the sample size is limited, and all participants are drawn from a single centre. The absence of specific clinical information for certain patients could introduce bias into this study. Secondly, there is a lack of sufficient experimental data to support the hypothesis based on our results. The findings of our research require validation through future multicentre prospective studies, and more monitoring indicators such as interleukin, complement C3/4. Due to the fact that TGP is a traditional Chinese medicine extract, related clinical trials are mostly conducted in China, and the level of evidence is not high. Therefore, multidimensional and large-scale clinical controlled trials are still needed to improve its evidence basis, and the observation indicators are often not comprehensive enough at present. Our hospital is the largest tertiary hospital in the province and can more comprehensively collect SS patients from all over the country.

## CONCLUSIONS

Through our study, the combined application can improve the exocrine gland function of patients with SS, inhibit excessive activation of the immune response, block the release of inflammatory immune factors, repair damaged blood systems, control disease activity, and have good clinical efficacy and safety with a higher quality life of patients. Thus, it provides reference for clinical physicians to treat SS, lay the foundation for subsequent clinical research.

### Author’s Contribution:

**TXF:** Conceived, designed and did statistical analysis & editing of manuscript, is responsible for integrity of research. **WWL and DBW:** Did data collection, statistical analysis and manuscript modification. **GLW:** Literature search, did review and final approval of manuscript.

## References

[1] Baldini C, Fulvio G, La Rocca G, Ferro F (2024). Update on the pathophysiology and treatment of primary Sjögren syndrome. Nat Rev Rheumatol.

[2] Van Beers JJBC, Damoiseaux JGMC (2021). Immune Monitoring upon Treatment with Biologics in Sjogren's Syndrome:The What, Where, When, and How. Biomolecules.

[3] Vivino FB, Carsons SE, Foulks G, Daniels TE, Parke A, Brennan MT (2016). New Treatment Guidelines for Sjögren's Disease. Rheum Dis Clin North Am.

[4] Bodard Q, Carre D, Chenal P, Zarnitsky C, Midhat M, Litrowski N (2020). Drug-induced Sweet's syndrome related to hydroxychloroquine:About 2 cases. Rev Med Interne.

[5] Lenfant T, Leroux G, Costedoat-Chalumeau N (2021). Different Control Populations May Lead to Different Understanding of Hydroxychloroquine Blood Levels as a Risk Factor for Retinopathy:Comment on the Article by Petri. Arthritis Rheumatol.

[6] Zhang L, Wei W (2020). Anti-inflammatory and immunoregulatory effects of paeoniflorin and total glucosides of paeony. Pharmacol Ther.

[7] Wu GL, Pu XH, Yu GY, Li TY (2015). Effects of total glucosides of peony on AQP-5 and its mRNA expression in submandibular glands of NOD mice with Sjogren's syndrome. Eur Rev Med Pharmacol Sci.

[8] Wu GL, Wu NY, Li TY, Lu WW, Yu GY (2016). Total glucosides of peony ameliorates sjgren's syndrome by affecting TH1/TH2 cytokine balance. Exp Ther Med.

[9] Lu WW, Fu TX, Wang Q, Chen YL, Li TY, Wu GL (2020). The effect of total glucoside of paeony on gut microbiota in NOD mice with Sjögren's syndrome based on high-throughput sequencing of 16SrRNA gene. Chin Med.

[10] Liu X, Li X, Li X, Li Z, Zhao D, Liu S (2019). The efficacy and safety of total glucosides of peony in the treatment of primary Sjögren's syndrome:a multi-center, randomized, double-blinded, placebo-controlled clinical trial (Correction appears in Clin Rheumatol 2019;(2)617-618. doi:10.1007/s10067-018-4378-6). Clin Rheumatol.

[11] Feng Z, Zhang BQ, Zhu YM, Yu BB, Fu L, Zhou LL (2019). The Effectiveness and Safety of Total Glucosides of Paeony in Primary Sjögren's Syndrome:A Systematic Review and Meta-Analysis. Front Pharmacol.

[12] Dai F, Yang G, Rao P, Wu P, Chen R, Sun Y (2020). Clinical Characteristics of Secondary Immune Thrombocytopenia Associated With Primary Sjögren's Syndrome. Front Med (Lausanne).

[13] Tasaki S, Suzuki K, Nishikawa A, Kassai Y, Takiguchi M, Kurisu R (2017). Multiomic disease signatures converge to cytotoxic CD8 T cells in primary Sjögren's syndrome. Ann Rheum Dis.

[14] Chen C, Liang Y, Zhang Z, Zhang Z, Yang Z (2020). Relationships between increased circulating YKL-40, IL-6 and TNF-αlevels and phenotypes and disease activity of primary Sjögren's syndrome. Int Immunopharmacol.

[15] Huo R, Wei C, Yang Y, Lin J, Huang X (2025). Hydroxychloroquine:A double-edged sword (Review). Mol Med Rep.

[16] Shiboski CH, Shiboski SC, Seror R, Criswell LA, Labetoulle M, Lietman TM (2017). 2016 American College of Rheumatology/European League Against Rheumatism Classification Criteria for Primary Sjögren's Syndrome:A Consensus and Data-Driven Methodology Involving Three International Patient Cohorts. Arthritis Rheumatol.

[17] Liu Y, Wang Z, Ren L, Zeng Q, Wang Z, Bian W (2022). Sonographic findings of immunoglobulin G4-related sialadenitis and differences from Sjögren's syndrome. Scand J Rheumatol.

[18] Bai W, Yang F, Xu H, Wei W, Li H, Zhang L (2023). A multi-center, open-label, randomized study to explore efficacy and safety of baricitinib in active primary Sjogren's syndrome patients [published correction appears in Trials.

[19] Iwata N, Tomiita M, Kobayashi I, Inoue Y, Nonaka Y, Okamoto N (2020). Utility of the EULAR Sjögren syndrome disease activity index in Japanese children:a retrospective multicenter cohort study. Pediatr Rheumatol Online J.

[20] Risselada AP, Kruize AA, Bijlsma JW (2012). Clinical applicability of the EULAR Sjogren's syndrome disease activity index:a cumulative ESSDAI score adds in describing disease severity. Ann Rheum Dis.

[21] Relangi HSK, Naidu GSRSNK, Sharma V, Kumar M, Dhir V, Sharmaet SK (2022). Association of immunological features with clinical manifestations in primary Sjogren's syndrome:a single-center cross-sectional study. Clin Exp Med.

